# Referral pattern to nephrologist and prognosis in diabetic kidney disease patients: Single center retrospective cohort study

**DOI:** 10.1371/journal.pone.0282163

**Published:** 2023-02-24

**Authors:** Yukimasa Iwata, Terumasa Hayashi, Hiroki Okushima, Ryuta Uwatoko, Taisuke Takatsuka, Daisuke Yoshimura, Tomohiro Kawamura, Rei Iio, Yoshiyasu Ueda, Tatsuya Shoji, Yoshitaka Isaka

**Affiliations:** 1 Department of Kidney Disease and Hypertension, Osaka General Medical Center, Osaka, Japan; 2 Department of Nephrology, Osaka University Graduate School of Medicine, Osaka, Japan; University of Campania Luigi Vanvitelli: Universita degli Studi della Campania Luigi Vanvitelli, ITALY

## Abstract

**Background:**

Management of diabetic kidney disease (DKD) to prevent end-stage kidney disease (ESKD) has become a major challenge for health care professionals. This study aims to investigate the characteristics of patients with DKD when they are first referred to a nephrologist and the subsequent prognoses.

**Methods:**

A total of 307 patients who were referred to our department from October 2010 to September 2014 at Osaka General Medical Center were analyzed. Independent risk factors associated with renal replacement therapy (RRT) and cardiovascular composite events (CVE) following their nephrology referral were later identified using Cox proportional hazards analysis.

**Results:**

Of 307 patients, 26 (8.5%), 67 (21.8%), 134 (43.6%), and 80 (26.1%) patients were categorized as having chronic kidney disease (CKD) stages 3a, 3b, 4, and 5, respectively. The median estimated glomerular filtration rate (eGFR) and urinary protein levels were 22.3 mL/min/1.73 m^2^ and 2.83 g/gCr, respectively, at the time of the nephrology referral. During the follow-up period (median, 30 months), 121 patients required RRT, and more than half of the patients with CKD stages 5 and 4 reached ESKD within 60 months following their nephrology referral; 30% and <10% of the patients with CKD stages 3b and 3a, respectively, required RRT within 60 months following their nephrology referral.

**Conclusion:**

Patients with DKD were referred to nephrologist at CKD stage 4. Although almost half of the patients with CKD stage 5 at the time of nephrology referral required RRT within one-and-a-half years after the referral, kidney function of patients who were referred to nephrologist at CKD stage 3 and 4 were well preserved.

## Introduction

Diabetic kidney disease (DKD) is the most common cause of end-stage kidney disease (ESKD), accounting for 42.5% of the patients who started chronic dialysis in 2017, according to the registry of the Japanese Society for Dialysis Therapy [1]. On the other hand, most of diabetic patients do not develop ESKD, as they die before [2]. Therefore, management of DKD to prevent ESKD and improve overall survival has become a major challenge in public health and clinical medicine.

Although several guidelines recommend that patients with CKD should be referred to a nephrologist at the CKD stage G4 [3–6], a large proportion of patients with CKD are still referred to nephrologists in the late phase of CKD in real-world clinical settings [7, 8].

Moreover, previous reports have shown that early referral to nephrologists is crucial for improving the mortality and morbidity rates in patients on chronic dialysis [9–18]. Although multifactorial intervention significantly reduced the onset or progression of DKD in patients with type 2 diabetes mellitus [19], patients with advanced DKD, despite the worst renal and cardiovascular prognosis, are at high risk of being under-treated independently of the type of clinical setting (nephrology, diabetology and primary care) [20].

There are few reports concerning patient characteristics with DKD referred to nephrologists for the first time and the prognosis thereafter [21].

Thus, the first aim of our study was to investigate the characteristics of patients with DKD at their first nephrologist referral. The second aim of our study was to examine the renal outcomes, all-cause mortality, and cardiovascular events after nephrology referral as well as to identify the factors associated with outcomes.

## Methods

### Patients

This study was conducted in accordance with the guidelines of the Declaration of Helsinki. This study was approved by the ethical committees of Osaka General Medical Center (No. 29-S1001).

All data were fully anonymized before we accessed them and ethics committee waived the requirement for informed consent. We clearly informed the patients and their relatives that the opportunity to opt out was always available on our hospital website.

**Fig 1:**
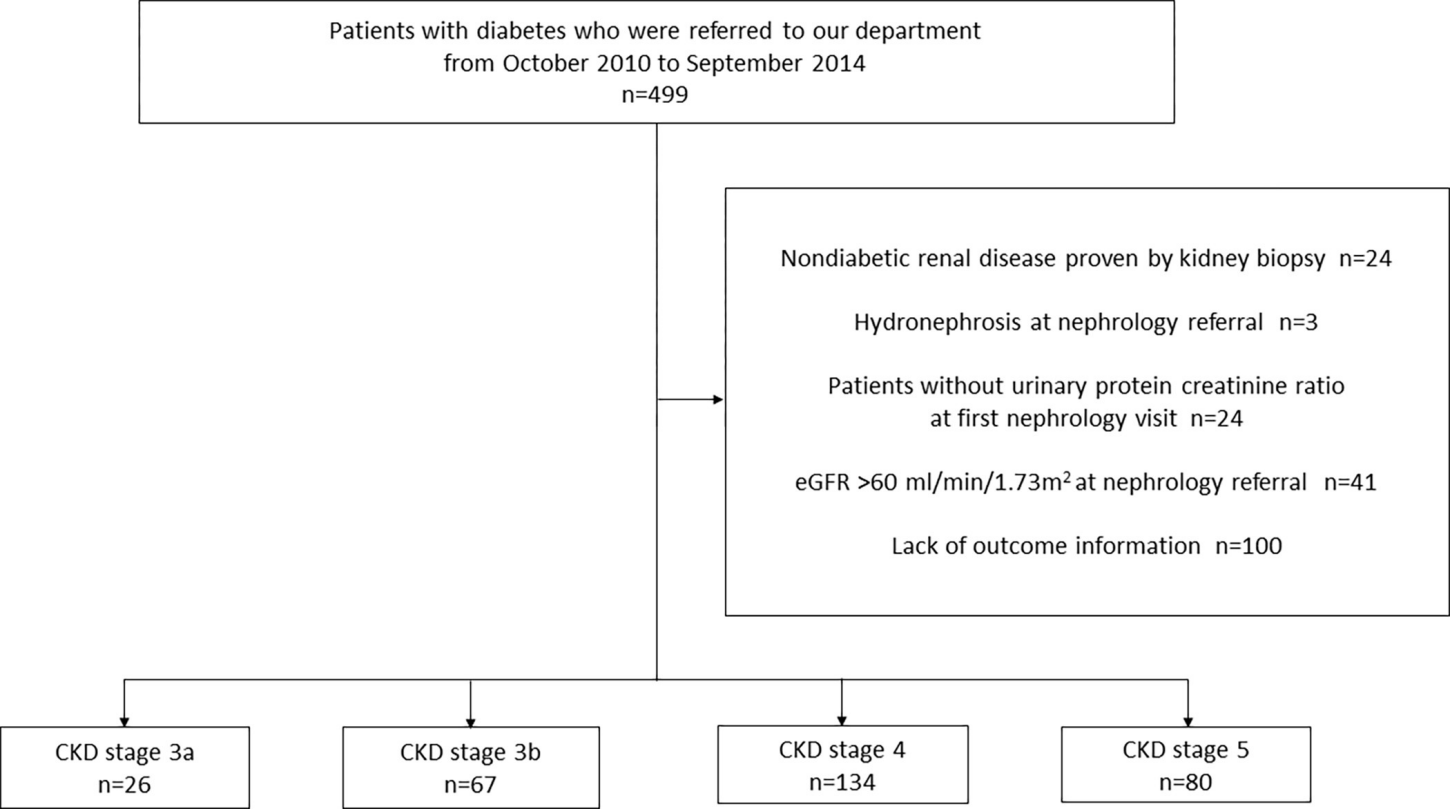
The flow chart of the study population.

This was a single-center, retrospective cohort study. A total of 499 pre-dialysis patients with CKD and diabetes mellitus, who were referred to our department between October 2010 and September 2014, were enrolled; further, 92 patients were excluded from the analysis owing to nondiabetic renal disease confirmed by renal biopsy (n = 24) or hydronephrosis (n = 3), at the first nephrology referral. Those who did not have data on urinary protein creatinine ratio at their first nephrology visit (n = 24) and those with eGFR >60 mL/min/1.73 m^2^ (n = 41) were also excluded. Further, 131 patients were referred back to their primary physicians because they were found to be at a low risk for progression to ESKD, as confirmed by the examining nephrologists. Therefore, questionnaires on the outcomes were sent to the primary physicians; however, outcome information was available for only 31 patients. We excluded 100 patients owing to a lack of data on their outcomes. In total, 307 patients were retrospectively studied (Fig 1).

### Data collection and definition

We collected the data of 307 patients from the electronic medical charts at Osaka General Medical Center, including demographics (sex and age), duration of diabetes mellitus, medications, comorbid conditions (history of cardiovascular disease, stroke, peripheral arterial disease, malignancy, and diabetic retinopathy), and clinical and laboratory variables (body mass index; blood pressure; heart rate; levels of serum creatinine, blood urea nitrogen, uric acid, hemoglobin, albumin, calcium, phosphate, intact parathyroid hormone, total cholesterol, low-density lipoprotein cholesterol, and glycated hemoglobin; and proteinuria). The glomerular filtration rate, at the nephrology referral, was estimated using the formula for Japanese people (eGFR[mL/min/1.73m2]=194×serumcreatinine−1.094×age−0.287×0.739[iffemales]) [22]. The patients were divided into four groups according to their renal function and CKD category (G3a-G5).

### Outcomes

The outcomes of interest in the present study were time to renal replacement therapy (RRT) and cardiovascular composite endpoints (CVE). CVE included all-cause death, coronary or peripheral revascularization, heart failure, or stroke. Patients were followed up after RRT initiation to evaluate the risk of CVE. RRT was defined as the requirement for maintenance dialysis or kidney transplantation. The index date was the day of the nephrology referral. The observation period lasted until December 2016. The median follow-up durations were 30 months (range, 11–43 months) and 35 months (range, 16.5–51 months) for RRT and CVE, respectively.

### Statistical analyses

Continuous variables are presented as median [interquartile range (IQR]) and were compared using the Kruskal-Wallis test. Categorical variables are presented as numbers and percentages and were compared using Fisher’s exact test. The incidence rates of RRT and CVE were compared using Kaplan–Meier curves and log-rank tests. Cox proportional hazards model was used to estimate the risk of RRT and CVE using hazard ratios (HR) and 95% confidence intervals (CIs). A competing risk model was used to analyze the risk of two event types: RRT and death.

Furthermore, multivariate Cox proportional hazards model was used to identify independent risk factors associated with the outcomes. Independent variables associated with the outcome in univariate analysis (P<0.1) were included in the multivariate analysis. Statistical significance was set at P <0.05. All statistical analyses were performed using the EZR software (Saitama Medical Center, Jichi Medical University, Saitama, Japan) [23].

## Results

### Baseline characteristics

At the nephrology referral, 26 (8.5%), 67 (21.8%), 134 (43.6%), and 80 (26.1%) patients were categorized as having CKD stages 3a, 3b, 4, and 5, respectively (Fig 1). Patient characteristics and baseline laboratory data for each group are listed in Table 1.

**Table 1:** Patient characteristics and laboratory data at nephrology referral by CKD stage.

	All patients	CKD stage 3a	CKD stage 3b	CKD stage 4	CKD stage 5	p value
	n = 307	n = 26	n = 67	n = 134	n = 80	
Male (%)	217 (70.7)	23 (88.5)	51 (76.1)	95 (70.9)	48 (60.0)	0.025
Age	69 [59–76]	65 [58–74]	68 [59–78]	69 [60–76]	69.0 [59–77]	0.684
Duration of diabetes (year)	15.0 [7.0–22.0]	13.0 [6.0–20.5]	15.0 [6.0–23.0]	13.0 [6.8–20.0]	18.0 [10.0–25.0]	0.223
Comorbid condition						
Cardiovascular disease (%)	92 (30.0)	5 (19.2)	12 (17.9)	46 (34.3)	29 (36.2)	0.032
Coronary artery disease (%)	67 (21.8)	4 (15.4)	11 (16.4)	30 (22.4)	22 (27.5)	0.346
Heart failure (%)	58 (18.9)	4 (15.4)	3 (4.5)	29 (21.6)	22 (27.5)	0.003
Stroke (%)	57 (18.6)	5 (19.2)	15 (22.4)	22 (16.4)	15 (18.8)	0.785
Peripheral artery disease (%)	24 (7.8)	2 (7.7)	5 (7.5)	9 (6.7)	8 (10.0)	0.857
Malignant disease (%)	32 (10.4)	4 (15.4)	7 (10.4)	12 (9.0)	9 (11.2)	0.788
Diabetic retinopathy (%) n = 267	158 (59.2)	14 (60.9)	31 (54.4)	68 (57.6)	45 (65.2)	0.631
Clinical and laboratory variables						
Body mass index	24.7 [22.5–28.1]	24.5 [22.9–28.5]	25.4 [23.1–28.0]	24.7 [22.5–27.8]	24.2 [22.3–27.9]	0.817
Systolic blood pressure (mmHg)	141 [129–159]	143 [134–158]	141 [128–157]	142 [127–162]	143 [130–159]	0.872
Diastolic blood pressure (mmHg)	78 [67–89]	88 [71–95]	80 [72–89]	77 [64–87]	77 [67–84]	0.051
Pulse rate (bpm)	77 [68–88]	82 [73–86]	75 [69–87]	78 [69–89]	76 [67–88]	0.707
Creatinine (mg/dL)	2.23 [1.55–3.20]	1.15 [1.04–1.20]	1.46 [1.29–1.64]	2.32 [1.91–2.72]	4.06 [3.45–5.11]	<0.001
Estimated glomerular filtration rate (ml/min/1.73 m^2^)	22.3 [14.6–32.6]	51.5 [48.5–54.0]	36.1 [33.1–40.2]	21.4 [18.3–25.9]	11.5 [9.0–13.2]	<0.001
Blood urea nitrogen (mg/dL)	32 [24–46]	17 [14–20]	23 [20–28]	33 [28–40]	52 [42–62]	<0.001
Uric acid (mg/dL)	7.0 [6.0–8.4]	6.0 [5.3–6.9]	6.5 [5.5–7.8]	7.2 [6.3–8.4]	7.7 [6.4–9.4]	<0.001
Hemoglobin (g/dL)	11.1 [9.9–12.9]	13.4 [12.1–14.1]	12.6 [11.3–13.6]	10.8 [9.8–12.3]	10.1 [8.9–11.0]	<0.001
Albumin (g/dL)	3.5 [2.9–4.0]	3.5 [3.0–4.1]	4.0 [3.5–4.2]	3.5 [2.8–4.0]	3.3 [2.8–3.7]	<0.001
Corrected calcium (mg/dL)	9.3 [9.0–9.6]	9.4 [9.0–9.6]	9.3 [9.1–9.6]	9.3 [9.0–9.7]	9.1 [8.7–9.5]	0.062
Phosphate (mg/dL)	3.7 [3.2–4.5]	3.2 [3.0–3.6]	3.3 [2.9–3.8]	3.6 [3.2–4.1]	4.8 [4.0–5.2]	<0.001
Intact parathyroid hormone (pg/mL) n = 167	107.0 [67.0–194.1]	61.8 [50.1–70.8]	65.3 [58.5–92.5]	101.7 [72.7–156.4]	194.0 [107.0–296.8]	<0.001
Total cholesterol (mg/dL) n = 220	182 [157–229]	177 [151–225]	183 [157–229]	182 [159–232]	184 [152–222]	0.91
Low-density lipoprotein cholesterol (mg/dL) n = 204	103 [82–130]	89 [66–105]	112 [89–145]	106 [89–130]	103 [74–120]	0.059
HbA1c (%)	6.7 [6.0–7.5]	7.7 [6.8–8.9]	6.7 [6.2–7.6]	6.7 [6.0–7.4]	6.3 [5.9–7.1]	<0.001
Urine protein-to-creatinine ratio (g/gCr)	2.83 [0.59–6.82]	2.00 [0.47–3.74]	1.36 [0.35–4.09]	3.16 [0.53–7.31]	4.34 [1.38–8.31]	0.001
Medication						
Renin-angiotensin system inhibitor (%)	211 (68.7)	19 (73.1)	46 (68.7)	90 (67.2)	56 (70.0)	0.932
Insulin (%)	77 (25.1)	5 (19.2)	19 (28.4)	36 (26.9)	17 (21.2)	0.635
Statin (%)	112 (36.5)	11 (42.3)	22 (32.8)	49 (36.6)	30 (37.5)	0.849

Values are presented as median (interquartile range) or n (percentage).

The median age was 69 years, and 70.7% of the patients were men. The median eGFR was 22.3 mL/min/1.73 m^2^ and urinary protein level was 2.83 g/gCr. The prevalence of heart failure significantly increased in CKD stages 4 and 5 compared with that in CKD stage 3. As CKD progressed, uric acid, phosphate, intact parathyroid hormone, and urinary protein levels increased, while hemoglobin, serum albumin, corrected calcium, and glycated hemoglobin levels decreased.

Patients without outcome information were older and had low blood urea nitrogen, uric acid, phosphate, and intact parathyroid hormone levels associated with high eGFR; of note, the urinary protein levels were lower and the eGFR was significantly higher in these patients than in the analyzed patients (S1 Table).

### Renal outcome by CKD stage at nephrology referral

**Fig 2:**
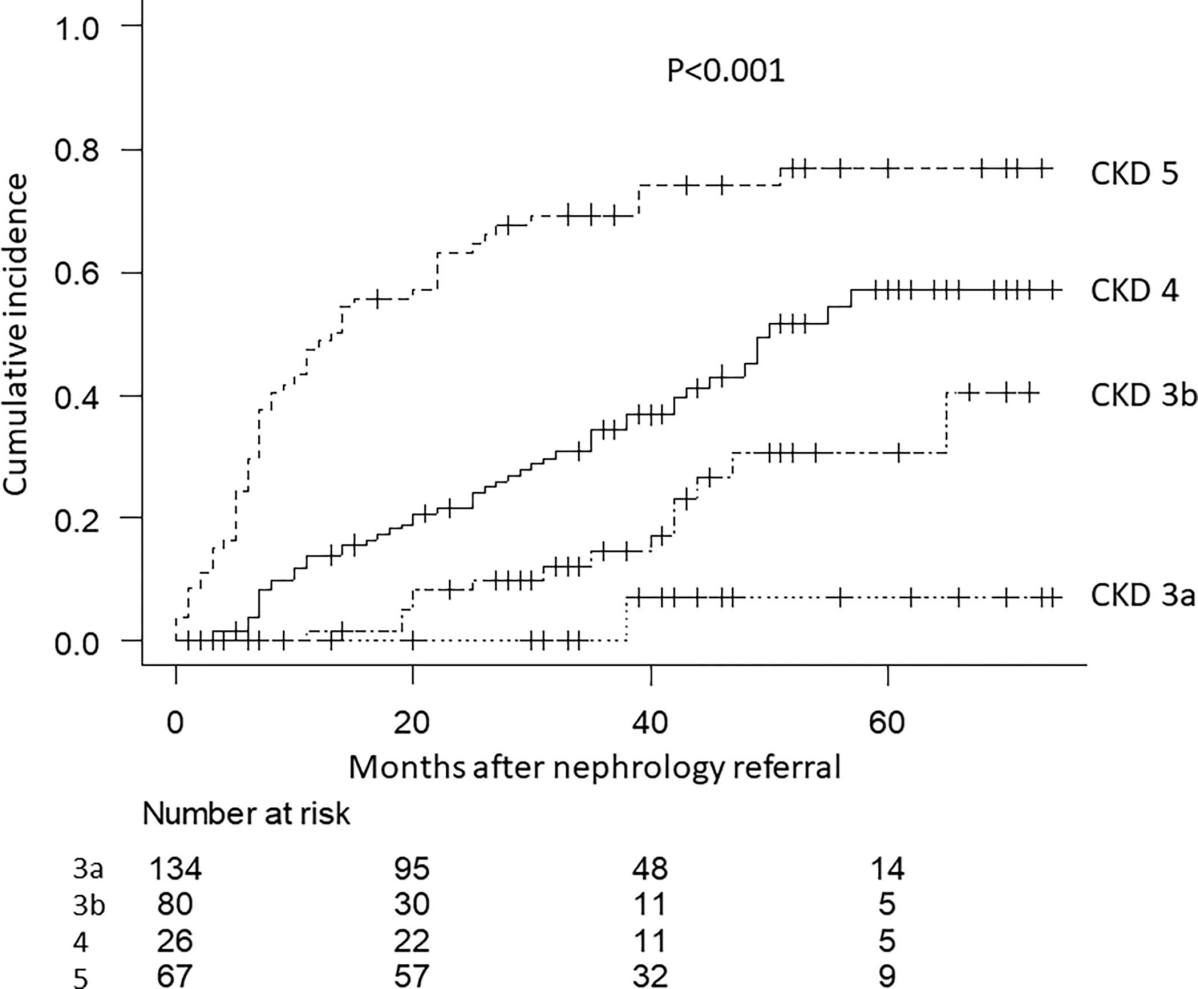
Renal outcome by CKD stage.

A total of 121 patients (39.4%) required RRT during the follow-up period. Kaplan–Meier analysis of renal outcomes showed a significant difference between the four groups (Fig 2). Moreover, almost half of the patients with CKD 5 reached ESKD within approximately 1 year after the nephrology referral.

Renal function at the nephrology referral was significantly associated with renal outcomes in both univariate [hazards ratio (HR), 0.91; 95% confidence interval (CI): 0.89–0.93; P<0.001) and multivariate Cox analyses (HR, 0.91; 95% CI: 0.88–0.94; P<0.001). Furthermore, young age (HR, 0.96; 95% CI: 0.95–0.98; P<0.001), history of malignancy (HR, 2.77; 95% CI, 1.48–5.16; P = 0.001), higher systolic blood pressure (HR, 1.01; 95% CI, 1.00–1.02; P = 0.026), and high serum albumin level (HR, 0.49; 95% CI: 0.34–0.71; P<0.001) were independently associated with renal outcomes in the multivariate analysis (Table 2).

**Table 2:** Indicators of renal outcome.

	Univariate analysis		Multivariate analysis	
	HR (95%CI)	p value	HR (95%CI)	p value
Male	0.97 (0.66–1.42)	0.860		
Age (year)	0.96 (0.95–0.97)	<0.001	0.96 (0.95–0.98)	<0.001
Diabetic duration (year)	0.99 (0.97–1.01)	0.274		
Cardiovascular disease	0.96 (0.65–1.43)	0.854		
Stroke	0.73 (0.44–1.21)	0.223		
Peripheral artery disease	0.83 (0.40–1.69)	0.602		
Malignancy	1.63 (0.93–2.86)	0.087	2.77 (1.48–5.16)	0.001
Body mass index	1.02 (0.98–1.06)	0.391		
Systolic blood pressure (mmHg)	1.01 (1.00–1.02)	0.005	1.01 (1.00–1.02)	0.026
Pulse rate (bpm)	1.01 (1.00–1.02)	0.246		
Estimated glomerular filtration rate (ml/min/1.73 m^2^)	0.91 (0.89–0.93)	<0.001	0.90 (0.87–0.93)	<0.001
Uric acid (mg/dL)	1.02 (0.93–1.12)	0.653		
Hemoglobin (g/dL)	0.81 (0.73–0.88)	<0.001	1.09 (0.97–1.22)	0.167
Albumin (g/dL)	0.38 (0.30–0.48)	<0.001	0.49 (0.34–0.71)	<0.001
Corrected calcium (mg/dL)	0.84 (0.63–1.11)	0.222		
Phosphate (mg/dL)	1.88 (1.59–2.23)	<0.001	1.18 (0.94–1.47)	0.159
HbA1c (%)	0.93 (0.79–1.09)	0.356		
Urine protein-to-creatinine ratio (g/gCr)	1.12 (1.09–1.14)	<0.001	1.03 (0.99–1.06)	0.114
Renin-angiotensin system inhibitor	1.33 (0.89–1.99)	0.166		
Insulin	1.48 (1.01–2.16)	0.043	1.42 (0.91–2.22)	0.125
Statin	1.10 (0.76–1.59)	0.597		

During the follow-up period, 35 patients died. Sensitivity analysis of the multivariate analysis showed that renal function at the nephrology referral was significantly associated with renal outcomes, using a competing risk model (HR, 0.91; 95% CI: 0.88–0.94; P<0.001). Furthermore, young age, history of malignancy, and low serum albumin levels were independently associated with renal outcomes in the multivariate analysis (S1 Fig and S2 Table).

### Association between CKD stage at nephrology referral and CVE

**Fig 3:**
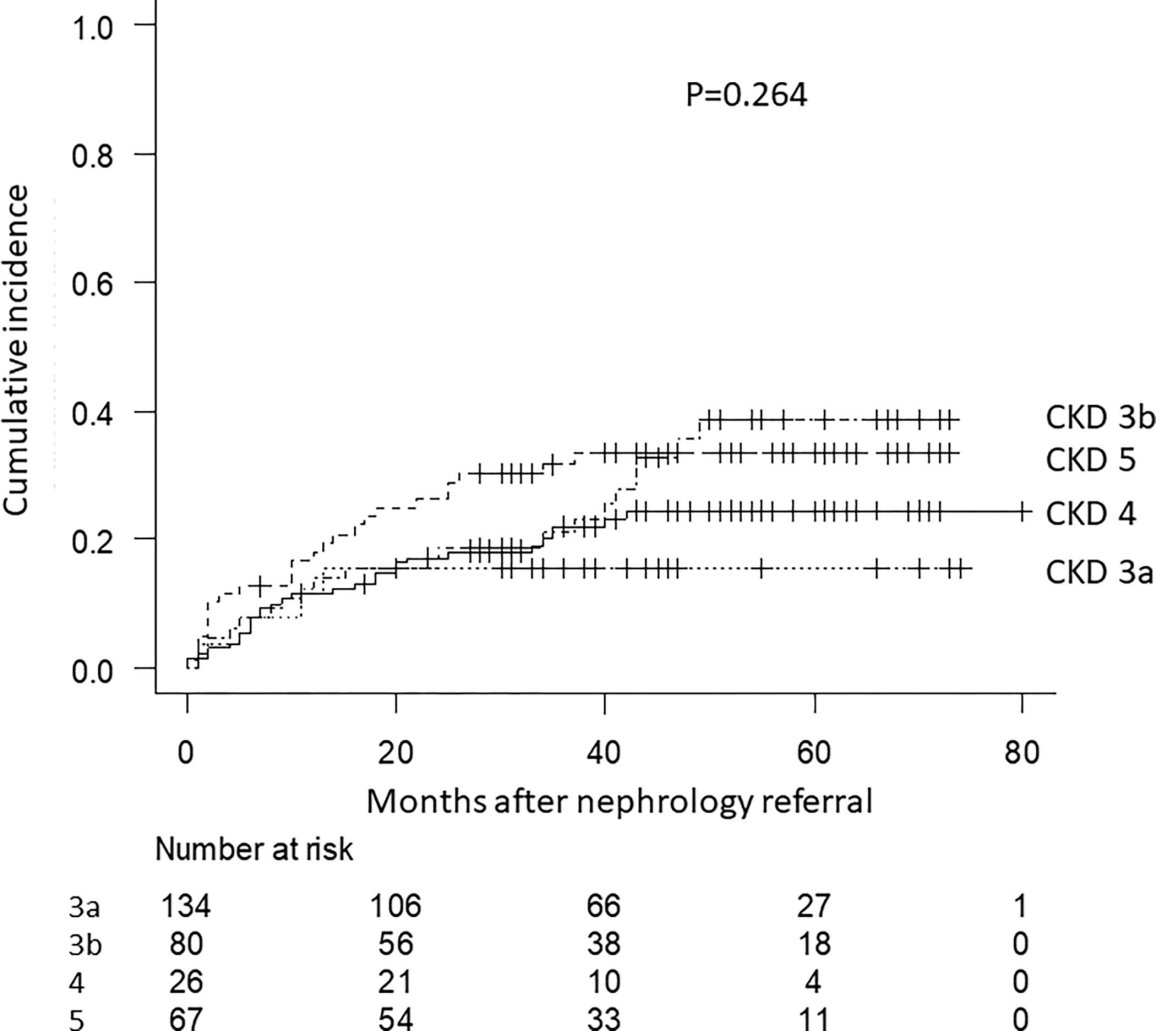
Cardiovascular composite outcome by CKD stage.

CVE occurred in 113 patients during the follow-up period, including 35 deaths from any cause, 21 from coronary artery disease, 39 from heart failure, 9 from peripheral artery disease, and 9 from stroke. Kaplan–Meier analysis of the cumulative incidence rate of CVE did not reveal a significant difference among the four groups (Fig 3). Renal function at the nephrology referral was not significantly associated with CVE (log-rank test, P = 0.264). As per the results of the multivariate Cox analysis for CVE, the significant variable was history of cardiovascular disease (HR, 1.64; 95% CI: 1.10–2.44; P = 0.015) (Table 3).

**Table 3:** Indicators of CVE.

	Univariate analysis		Multivariate analysis	
	HR (95%CI)	p value	HR (95%CI)	p value
Male	0.83 (0.56–1.23)	0.358		
Age (year)	1.03 (1.01–1.04)	0.003	1.02 (1.00–1.04)	0.068
Diabetic duration (year)	1.02 (1.00–1.03)	0.100		
Cardiovascular disease	2.03 (1.40–2.96)	<0.001	1.64 (1.10–2.44)	0.015
Stroke	1.41 (0917–2.19)	0.123		
Peripheral artery disease	1.67 (0.94–2.98)	0.082	1.21 (0.66–2.22)	0.543
Malignancy	1.72 (1.01–2.92)	0.045	1.42 (0.82–2.45)	0.215
Body mass index	0.96 (0.92–1.00)	0.063	0.97 (0.93–1.02)	0.226
Systolic blood pressure (mmHg)	1.00 (0.99–1.01)	0.437		
Pulse rate (bpm)	1.00 (0.99–1.01)	0.980		
Estimated glomerular filtration rate (ml/min/1.73 m^2^)	0.99 (0.97–1.01)	0.189		
Uric acid (mg/dL)	1.02 (0.93–1.12)	0.714		
Hemoglobin (g/dL)	0.94 (0.85–1.03)	0.168		
Albumin (g/dL)	0. 83 (0.65–1.06)	0.133		
Corrected calcium (mg/dL)	1.00 (0.76–1.32)	0.989		
Phosphate (mg/dL)	1.18 (0.96–1.44)	0.108		
HbA1c (%)	1.11 (0.95–1.28)	0.182		
Urine protein-to-creatinine ratio (g/gCr)	1.01 (0.98–1.05)	0.519		
Renin-angiotensin system inhibitor	0.86 (0.58–1.28)	0.457		
Insulin	0.98 (0.64–1.50)	0.911		
Statin	1.39 (0.96–2.03)	0.084	1.35 (0.92–1.98)	0.129

## Discussion

Timely referral to a nephrologist and adequate care of patients with CKD are important aspects for improving clinical outcomes. The benefits of early referral to a nephrologist have been well-established in previous studies [9–18, 24]. Several guidelines recommend early nephrology referral, especially for certain patients whose renal function declined rapidly, such as those with DKD [5, 6].

Although many clinicians recognize the importance of timely nephrology referral in patients with CKD, a large proportion of CKD patients are still referred late in the real-world clinical settings [7, 8], and there are few reports on the characteristics of DKD patients at the nephrology referral and their outcomes. The present study showed that 70% of the patients with DKD were referred to a nephrologist at CKD stage 4 or 5.

Pinier et al. reported that the eGFR at nephrology referral declined to 42.2 mL/min/1.73 m^2^ in DKD patients, whose mean age was 70.3 years. In this study, 26.3% of the patients were referred to a nephrologist for reasons other than decreased renal function, albuminuria, and hypertension [25] indicating that renal function in these patients may be maintained.

Because the patients in our study were referred to our department mainly for declined renal function and albuminuria, the renal function of our patients had declined to 22.3 mL/min/1.73 m^2^ at the time of referral.

Although more than half of the patients with CKD stages 5 and 4 reached ESKD within 60 months after the nephrology referral, 30% and <10% of the patients, with CKD stages 3b and 3a, respectively required RRT within 60 months. The renal outcomes in our study were worse than those reported in previous studies, such as RENAAL and IDNT [26, 27]. The reason for this may be that patients with a low risk of progression to ESKD after examination by nephrologists were referred back to their primary physicians, and of the 131 patients, we could investigate only 31 patients’ outcomes.

Our results suggest that nephrology referral until CKD stage 3a is important for DKD patients, and these patients should be referred to a nephrologist until CKD stage 3b even if their renal function declines to less than eGFR 45 mL/min/1.73 m^2^.

In previous reports, the eGFR of patients with type 1 and 2 diabetes declined to 9.1 and 0.5–1.9 mL/min/1.73 m^2^/year, respectively [28, 29]. The renal function of our patients declined rapidly because they had higher levels of proteinuria than those reported in previous reports. It was also reported that diabetic retinopathy could predict subsequent cardiovascular events in patients with diabetes mellitus [30]; however, due to the retrospective nature of this study, we didn’t have enough data on diabetic retinopathy, so we could not investigate the association between diabetic retinopathy and CVE onset.

Renin-angiotensin system inhibitors have been found to reduce urinary protein levels and improve renal outcomes in patients with DKD [26, 27]. Nephrological care, including low-protein diet, appropriate blood pressure control, and use of renin-angiotensin system inhibitors, is useful for decreasing urinary protein excretion. Martínez-Ramírez et al. showed that nephrologists prescribed renin-angiotensin system inhibitors more frequently than family physicians, leading to increased renal function preservation in patients with diabetes mellitus [31].

In our study, urinary protein excretion was not independently associated with renal outcomes in the multivariate analysis but lower serum albumin associated with renal outcomes. We measured urinary protein excretion as spot urine protein-creatinine ratio and it is already known that spot urine albumin-creatinine ratio had day-to-day variability [32]. In addition, the number of RRT was relatively small. For above reasons we could not find the significant relation between renal outcomes and urinary protein excretion.

Renal function, albuminuria, and history of cardiovascular disease have been found to be associated with the incidence of cardiovascular disease in patients with CKD in previous reports [33–35].

In the present study, history of cardiovascular disease was associated with CVE in the multivariate analysis, but renal function and urinary protein excretion were not. One of the considerable reasons is that the number of CVE is small in the present study and it is reported that incidences of cardiovascular disease in CKD patients is lower in Japan compared to those in other countries [36].

We previously reported that more than 20 months of pre-dialysis nephrological care may be needed to improve the first-year mortality after dialysis initiation [18]. However, Fig 2 shows that more than 50% of the patients with CKD 5 and almost 20% with CKD 4 required RRT within 20 months of the nephrology referral. Previous reports have shown that in patients with DKD early nephrology referral not only preserves renal function but is also associated with better overall survival even if the patients need chronic dialysis treatment [25, 31].

Moreover, recently, new renoprotective agents have been developed for the treatment of DKD, such as sodium glucose cotransporter 2 (SGLT2) inhibitors, selonsertib, finerenone, and atrasentan [37–43]. However, the target patients in those clinical trials were restricted to those with eGFR of 20–25 mL/min/1.73 m^2^ or more. In other words, the impact of these agents on the advanced stages of DKD has not been revealed. Thus, early referral to nephrologists is necessary to slow the progression of DKD with the help of both renin-angiotensin system inhibitors and these new agents.

In addition to renoprotective effects, these new agents, such as SGLT2 inhibitors and finerenone, improve the cardiovascular outcomes [38, 42, 44, 45]. However, because these studies excluded patients with eGFR of 25–30 mL/min/1.73 m^2^ or less and furthermore, in the present study, SGLT2 inhibitors were not widely used, so it is not clear whether these agents will improve the cardiovascular outcomes in patients with advanced stages of DKD.

The present study had some limitations. First, this study was a single-center retrospective cohort study; however, the inclusion of a relatively large number of study patients, collection of data from electronic medical charts, and high-quality outcome survey improved our analyses.

Second, we did not investigate the treatment details after the nephrology referral, such as blood pressure, fluid and mineral levels, bone disorders, anemia, metabolic acidosis, glycemic control and medications.

Third, we did not investigate if the patients received primary care before the nephrology referral. Some patients who did not receive primary care visited the emergency room with advanced CKD at the last moment, requiring emergency dialysis.

Fourth, we did not consider the effects of dialysis initiation, including medication use and dialysis efficiency. The patients in the present study were similarly managed at various dialysis centers based on several clinical guidelines.

Fifth, our hospital is a tertiary hospital; therefore, the prevalence of patients with multiple and severe comorbidities, including cardiovascular disease and malignancy, was extremely high. This may have affected the results of this study.

## Conclusion

Our results suggest that the eGFR in patients with DKD had declined to 22.3 mL/min/1.73 m^2^ at their nephrology referral and almost half of the patients with CKD stage 5 at the referral required RRT within one-and-a-half year after the referral, while kidney function in patients with CKD stages 3 and 4 was well preserved. Further studies are needed to identify the optimal timing for nephrology referral to improve the prognosis of patients with DKD.

## Supporting information

S1 Fig Renal outcome by CKD stage (competing risk model). (TIF)

S1 Table Patient characteristics and laboratory data at nephrology referral compared to excluded patients. (TIF)

S2 Table Indicators of renal outcome (competing risk model). (TIF)
