# Supplementary figures and images for: Referral pattern to nephrologist and prognosis in diabetic kidney disease patients: Single center retrospective cohort study
Source: PLoS One. 2023 Feb 24;18(2):e0282163. doi: 10.1371/journal.pone.0282163 (PMC9956043; doi:10.1371/journal.pone.0282163)

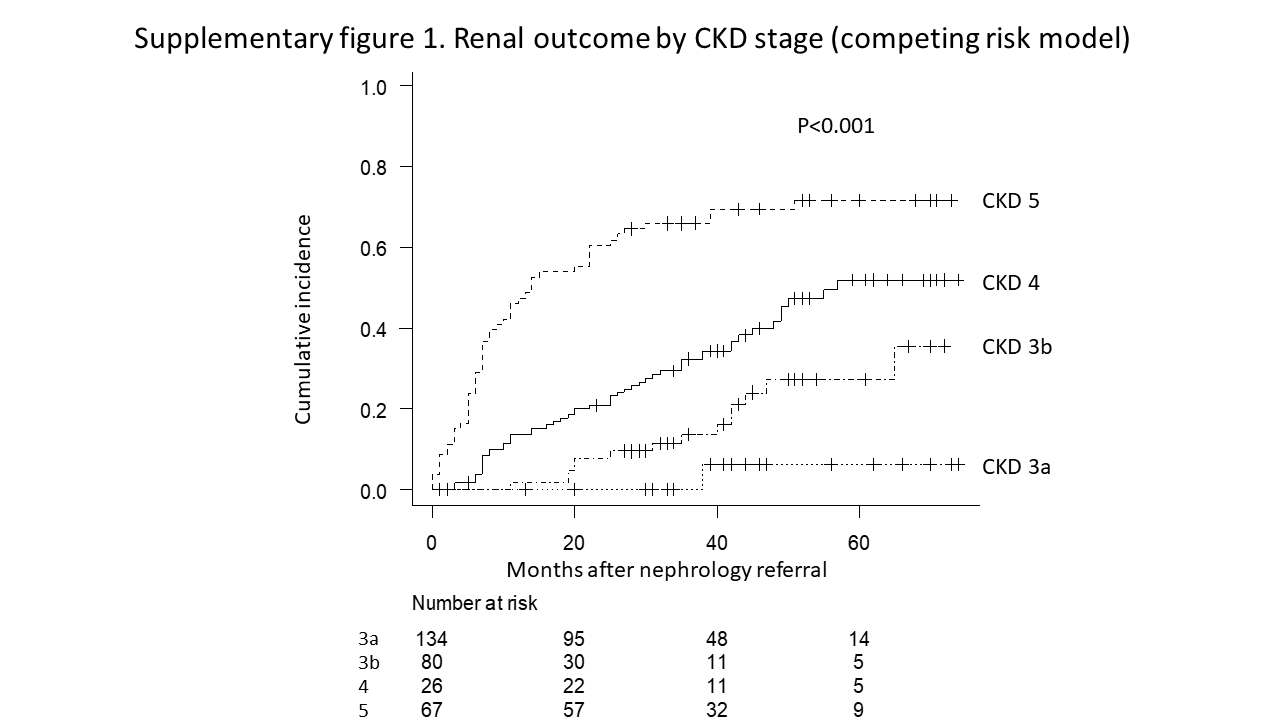

Supplement: S1 Fig — (TIF) [file pone.0282163.s001.tif]

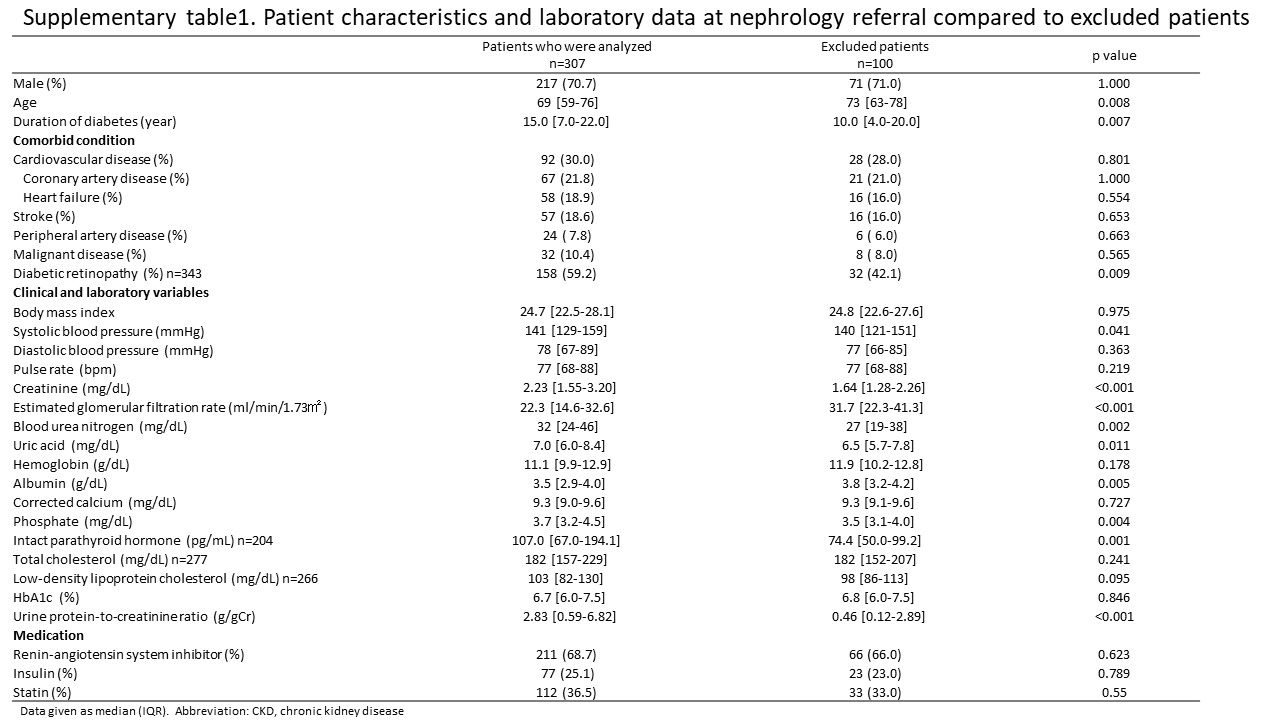

Supplement: S1 Table — (TIF) [file pone.0282163.s002.tif]

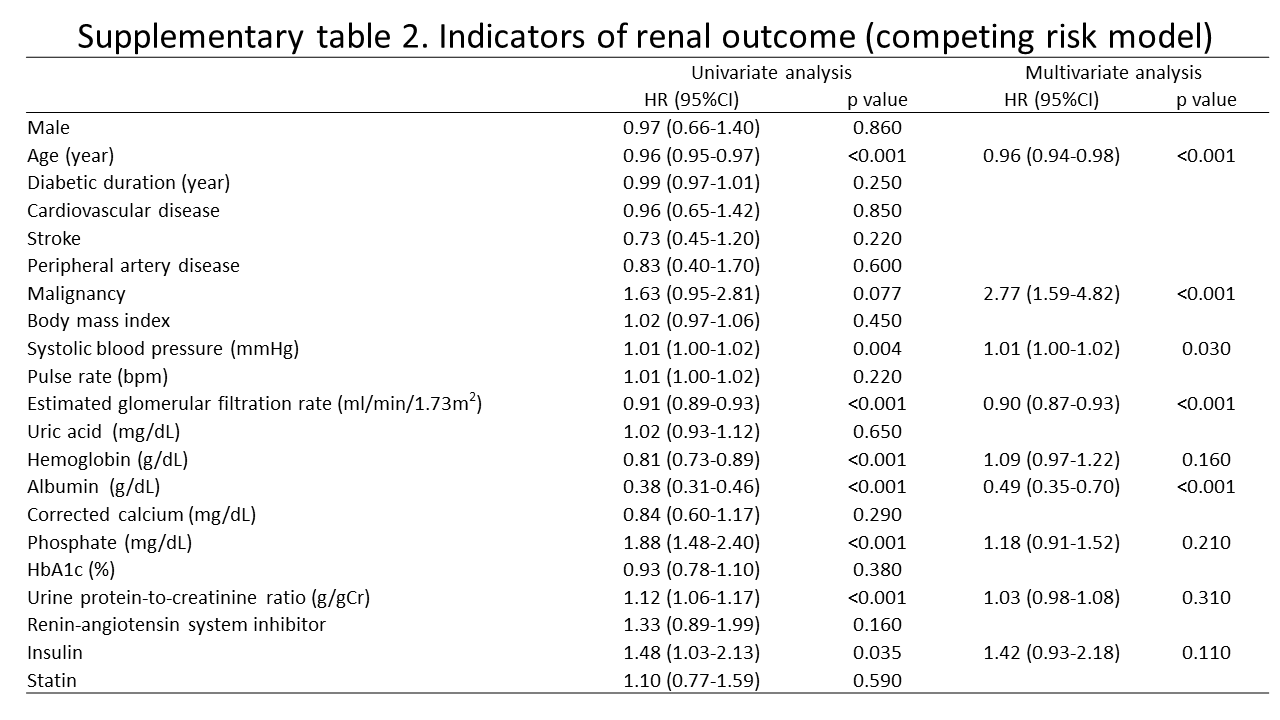

Supplement: S2 Table — (TIF) [file pone.0282163.s003.tif]
