# Supplementary material for: Referral pattern to nephrologist and prognosis in diabetic kidney disease patients: Single center retrospective cohort study
Source: PLoS One. 2023 Feb 24;18(2):e0282163. doi: 10.1371/journal.pone.0282163 (PMC9956043; doi:10.1371/journal.pone.0282163)
Supplement: Attachment — Submitted filename: Response to Reviewers.docx [file pone.0282163.s004.docx]

Dr. Ferdinando Carlo Sasso

Academic Editor

Plos one

*December 1, 2022*

Dear Dr *Sasso*,

We wish to express our strong appreciation to the reviewers for their insightful comments on our paper entitled, “Referral pattern to nephrologist and prognosis in diabetic kidney disease patients: single center retrospective cohort study.”. We feel the comments have helped us significantly improve the manuscript.

We have incorporated changes that reflect the detailed suggestions you have graciously provided.

We have marked the changes highlighted in yellow in the text of the revised paper.

We hope that the revisions in the manuscript and our accompanying responses will be sufficient to make our manuscript suitable for publication in Plos one.

Journal Requirements:

1. Please ensure that your manuscript meets PLOS ONE's style requirements, including those for file naming. The PLOS ONE style templates can be found at
   <https://journals.plos.org/plosone/s/file?id=wjVg/PLOSOne_formatting_sample_main_body.pdf> and 
   <https://journals.plos.org/plosone/s/file?id=ba62/PLOSOne_formatting_sample_title_authors_affiliations.pdf>

Thank you for your comments. We modified our manuscript and supportive files.

2. Please amend your current ethics statement to address the following concerns:
   a) Did participants provide their written or verbal informed consent to participate in this study?
   b) If consent was verbal, please explain i) why written consent was not obtained, ii) how you documented participant consent, and iii) whether the ethics committees/IRB approved this consent procedure.
   c) If you are reporting a retrospective study of medical records or archived samples, please ensure that you have discussed whether all data were fully anonymized before you accessed them and/or whether the IRB or ethics committee waived the requirement for informed consent. If patients provided informed written consent to have data from their medical records used in research, please include this information.

We appreciate your comment on this point. Accordingly, we have added the following text to the Method section (page. 5, line 4): All data were fully anonymized before we accessed them and ethics committee waived the requirement for informed consent.

3. Thank you for stating the following in the Acknowledgments Section of your manuscript:

"This research was supported in part by grant from the Osaka Kidney Foundation (OKF18-0001)."

We note that you have provided funding information that is not currently declared in your Funding Statement. However, funding information should not appear in the Acknowledgments section or other areas of your manuscript. We will only publish funding information present in the Funding Statement section of the online submission form. 
Please remove any funding-related text from the manuscript and let us know how you would like to update your Funding Statement. Currently, your Funding Statement reads as follows:

"Y.I received grant from Osaka Kidney Foundation (<http://www.okf.ecnet.jp/>). Grant number is OKF18-0001.

The funders had no role in study design, data collection and analysis, decision to publish, or preparation of the manuscript."

Please include your amended statements within your cover letter; we will change the online submission form on your behalf.

Thank you very much for your comments. We removed the Acknowledge section from manuscript. I would like to update my Finding statement as follows:

"Y.I. received grant from Osaka Kidney Foundation (<http://www.okf.ecnet.jp/>). Grant number is OKF18-0001.

The funders had no role in study design, data collection and analysis, decision to publish, or preparation of the manuscript."

4. PLOS requires an ORCID iD for the corresponding author in Editorial Manager on papers submitted after December 6th, 2016. Please ensure that you have an ORCID iD and that it is validated in Editorial Manager. To do this, go to ‘Update my Information’ (in the upper left-hand corner of the main menu), and click on the Fetch/Validate link next to the ORCID field. This will take you to the ORCID site and allow you to create a new iD or authenticate a pre-existing iD in Editorial Manager. Please see the following video for instructions on linking an ORCID iD to your Editorial Manager account: <https://www.youtube.com/watch?v=_xcclfuvtxQ>

Thank you for your comment. In accordance with your comment, we have ensured ORCID ID.

5. Please amend either the title on the online submission form (via Edit Submission) or the title in the manuscript so that they are identical.

We appreciate your suggestion. We have changed title as follows: Referral pattern to nephrologist and prognosis in diabetic kidney disease patients: single center retrospective cohort study

6. Please include captions for your Supporting Information files at the end of your manuscript, and update any in-text citations to match accordingly. Please see our Supporting Information guidelines for more information: <http://journals.plos.org/plosone/s/supporting-information>.

Thank you for your comments. Accordingly, we added Supporting information files at the end of our manuscript including captions.

7 Please review your reference list to ensure that it is complete and correct. If you have cited papers that have been retracted, please include the rationale for doing so in the manuscript text, or remove these references and replace them with relevant current references. Any changes to the reference list should be mentioned in the rebuttal letter that accompanies your revised manuscript. If you need to cite a retracted article, indicate the article’s retracted status in the References list and also include a citation and full reference for the retraction notice.

Thank you for your suggestion. We modified our reference list. We removed one reference paper: Imai E, et al. CKD clinical practice guideline 2012. Nihon Jinzo Gakkai shi. 2012; 54: 1031-191.

In addition, we added seven papers to reference section: Sasso FC, Pafundi PC, Simeon V, Nicola LD, Chiodini P, Galiero R, et al. Efficacy and durability of multifactorial intervention on mortality and MACEs: a randomized clinical trial in type-2 diabetic kidney disease. Cardiovasc Diabetol. 2021; 20: 145, Ueki K, Sasako T, Okazaki Y, Miyake K, Nangaku M, Ohashi Y, et al. Multifactorial intervention has a significant effect on diabetic kidney disease in patients with type 2 diabetes. Kidney Int. 2021; 99; 256-266, Minutolo R, Sasso FC, Chiodini P, Cianciaruso B, Carbonara O, Zamboli P, et al. Management of cardiovascular risk factors in advanced type 2 diabetic nephropathy: a comparative analysis in nephrology, diabetology and primary care settings. J Hypertens. 2006; 24; 1655-61, Sasso FC, Pafundi PC, Gelso A, Bono V, Costagliola C, Marfella R, et al. Relationship between albuminuric CKD and diabetic retinopathy in a real-world setting of type 2 diabetes: Findings from No blind study. Nutr Metab Cardiovasc Dis. 2019; 29: 923-930, Naresh CN, Hayen A, Weening A, Craig JC, Chadban SJ. Day-to-day variability in spot urine albumin-creatinine ratio. Am J Kidney Dis. 2013; 62: 1095-101, Tanaka K, Watanabe T, Takeuchi A, Ohashi Y, Nitta K, Akizawa T, et al. Cardiovascular events and death in Japanese patients with chronic kidney disease. Kidney Int. 2017; 91: 227-234 and Nevola R, Alfano M, Pafundi PC, Brin C, Gragnano F, Calabrò P, et al. Cardiorenal Impact of SGLT-2 Inhibitors: A Conceptual Revolution in The Management of Type 2 Diabetes, Heart Failure and Chronic Kidney Disease. Rev Cardiovasc Med. 2022; 23; 106.

Therefore, we correct the number of reference list.

Review Comments to the Author
Reviewer #2: I read with great interest the paper “Referral pattern to nephrologist and prognosis in diabetic kidney disease patients: single center retrospective cohort study " by Iwata et al.
The article is well written. Paper design is fine. The article is logically divided into sections and subsections.
Comments:
1. Introduction: Diabetic nephropathy should be addressed to the right specialist as soon as possible to start the appropriate therapy and follow-up, in order to slow the disease progression. However, previous data have reported that despite the worst renal and cardiovascular prognosis, this subset of patients are at high risk of being under-treated independently of the type of clinical setting (nephrology, diabetology and primary care) (doi: 10.1097/01.hjh.0000239303.93872.31). This is also in contrast with the new view point of the multifactorial intervention in order to reduce the end stage kidney disease progression, but, even more importantly the cardiovascular risk factors. In fact, most of diabetic patients do not develop end stage kidney disease, as they die before (doi: 10.1186/s12933-021-01343-1).

We appreciate your comments on this point. We have added and modified the following text to the introduction section (page. 3, line 14): On the other hand, most of diabetic patients do not develop ESKD, as they die before [2]. Therefore, management of DKD to prevent ESKD and improve overall survival has become a major challenge in public health and clinical medicine.

In addition, we have added the sentences in the introduction section (page. 4, line 6): Although multifactorial intervention significantly reduced the onset or progression of DKD in patients with type 2 diabetes mellitus [19], patients with advanced DKD, despite the worst renal and cardiovascular prognosis, are at high risk of being under-treated independently of the type of clinical setting (nephrology, diabetology and primary care) [20].

2. Discussion, line 25-27: the authors reported that in their population the proteinuria levels are higher than in previous study, with a rapid decline in renal function. In these patients I would have also expected an increase in diabetic retinopathy onset with a consequent increase in cardiovascular events (doi: 10.1016/j.numecd.2019.05.065). Moreover, the authors reported after a line that renin angiotensin system inhibitors are used in this patients to reduce proteinuria, which is true, but with the use of this drugs we first assist to a reduction in glomerular filtration rate and after a stabilization in time, which may be compatible with a rapid decline in kidney function. Moreover, the role of SGLT2 inhibitors has been spread in this population, with an amelioration of both proteinuria and kidney function, together with a reduction of cardiovascular events, also representing the future, and not reported in the article (doi: 10.31083/j.rcm2303106).

We appreciate your suggestion on this point. Regrettably, we did not retrieve the information from all patient’s records whether patients had the diabetic retinopathy or not because this is retrospective study. We added the sentences to the discussion section (page. 18, line 6); It was also reported that diabetic retinopathy could predict subsequent cardiovascular events in patients with diabetes mellitus [30]; however, due to the retrospective nature of this study, we didn't have enough data on diabetic retinopathy, so we could not investigate the association between diabetic retinopathy and CVE onset.

Although about 70% of our patients already used renin angiotensin system inhibitors at nephrology referral, we did not investigate the treatment details including medications after nephrology referral. So we modified the sentences in discussion section (page. 21, line 6): Second, we did not investigate the treatment details after the nephrology referral, such as blood pressure, fluid and mineral levels, bone disorders, anemia, metabolic acidosis, glycemic control and medications.

In response to reviewer’s comment, we modified the sentence in the discussion section (page. 20, lines 11); However, because these studies excluded patients with eGFR of 25–30 mL/min/1.73 m^2^ or less and furthemore, in the present study, SGLT2 inhibitors were not widely used, so it is not clear whether these agents will improve the cardiovascular outcomes in patients with advanced stages of DKD. Early nephrology referral may improve both renal and cardiovascular outcomes.

Yours sincerely,

*Yukimasa Iwata*

Department of Kidney Disease and Hypertension, Osaka General Medical Center

3-1-56 Bandaihigashi, Sumiyoshi-ku, Osaka 558-8558, Japan

Tel: *+81-6-6692-1201*

Fax: *+81-6-6606-7000*

E-mail: *yuki_iwata0718@yahoo.co.jp*
