# Supplementary material for: Referral pattern to nephrologist and prognosis in diabetic kidney disease patients: Single center retrospective cohort study
Source: PLoS One. 2023 Feb 24;18(2):e0282163. doi: 10.1371/journal.pone.0282163 (PMC9956043; doi:10.1371/journal.pone.0282163)
Supplement: Peer review history

# Peer review: Decision Letter 0

**DOI:** 10.1371/journal.pone.0282163.r001

**Authors:** Ferdinando Carlo Sasso

18 Jul 2022

PONE-D-22-18940Referral pattern to nephrologist and prognosis in diabetic kidney disease patients: single center retrospective cohort studyPLOS ONE

Dear Dr. Hayashi,

Thank you for submitting your manuscript to PLOS ONE. After careful consideration, we feel that it has merit but does not fully meet PLOS ONE’s publication criteria as it currently stands. Therefore, we invite you to submit a revised version of the manuscript that addresses the points raised during the review process.

Please submit your revised manuscript by two weeks. If you will need more time than this to complete your revisions, please reply to this message or contact the journal office at plosone@plos.org. When you're ready to submit your revision, log on to https://www.editorialmanager.com/pone/ and select the 'Submissions Needing Revision' folder to locate your manuscript file.

Please include the following items when submitting your revised manuscript:

- A rebuttal letter that responds to each point raised by the academic editor and reviewer(s). You should upload this letter as a separate file labeled 'Response to Reviewers'.
- A marked-up copy of your manuscript that highlights changes made to the original version. You should upload this as a separate file labeled 'Revised Manuscript with Track Changes'.
- An unmarked version of your revised paper without tracked changes. You should upload this as a separate file labeled 'Manuscript'.

If you would like to make changes to your financial disclosure, please include your updated statement in your cover letter. Guidelines for resubmitting your figure files are available below the reviewer comments at the end of this letter.

If applicable, we recommend that you deposit your laboratory protocols in protocols.io to enhance the reproducibility of your results. Protocols.io assigns your protocol its own identifier (DOI) so that it can be cited independently in the future. For instructions see: https://journals.plos.org/plosone/s/submission-guidelines#loc-laboratory-protocols. Additionally, PLOS ONE offers an option for publishing peer-reviewed Lab Protocol articles, which describe protocols hosted on protocols.io. Read more information on sharing protocols at https://plos.org/protocols?utm_medium=editorial-email&utm_source=authorletters&utm_campaign=protocols.

We look forward to receiving your revised manuscript.

Kind regards,

Ferdinando Carlo Sasso, PhD, MD

Academic Editor

PLOS ONE

Journal Requirements:

When submitting your revision, we need you to address these additional requirements.

1. Please ensure that your manuscript meets PLOS ONE's style requirements, including those for file naming. The PLOS ONE style templates can be found at 

https://journals.plos.org/plosone/s/file?id=wjVg/PLOSOne_formatting_sample_main_body.pdf and 

https://journals.plos.org/plosone/s/file?id=ba62/PLOSOne_formatting_sample_title_authors_affiliations.pdf

2. Please amend your current ethics statement to address the following concerns:

a) Did participants provide their written or verbal informed consent to participate in this study?

b) If consent was verbal, please explain i) why written consent was not obtained, ii) how you documented participant consent, and iii) whether the ethics committees/IRB approved this consent procedure.

c) If you are reporting a retrospective study of medical records or archived samples, please ensure that you have discussed whether all data were fully anonymized before you accessed them and/or whether the IRB or ethics committee waived the requirement for informed consent. If patients provided informed written consent to have data from their medical records used in research, please include this information.

3. Thank you for stating the following in the Acknowledgments Section of your manuscript: 

"This research was supported in part by grant from the Osaka Kidney Foundation (OKF18-0001)."

We note that you have provided funding information that is not currently declared in your Funding Statement. However, funding information should not appear in the Acknowledgments section or other areas of your manuscript. We will only publish funding information present in the Funding Statement section of the online submission form. 

Please remove any funding-related text from the manuscript and let us know how you would like to update your Funding Statement. Currently, your Funding Statement reads as follows: 

"Y.I received grant from Osaka Kidney Foundation (http://www.okf.ecnet.jp/). Grant number is OKF18-0001.

The funders had no role in study design, data collection and analysis, decision to publish, or preparation of the manuscript."

Please include your amended statements within your cover letter; we will change the online submission form on your behalf. 

4. PLOS requires an ORCID iD for the corresponding author in Editorial Manager on papers submitted after December 6th, 2016. Please ensure that you have an ORCID iD and that it is validated in Editorial Manager. To do this, go to ‘Update my Information’ (in the upper left-hand corner of the main menu), and click on the Fetch/Validate link next to the ORCID field. This will take you to the ORCID site and allow you to create a new iD or authenticate a pre-existing iD in Editorial Manager. Please see the following video for instructions on linking an ORCID iD to your Editorial Manager account: https://www.youtube.com/watch?v=_xcclfuvtxQ

5. Please amend either the title on the online submission form (via Edit Submission) or the title in the manuscript so that they are identical.

6. Please include captions for your Supporting Information files at the end of your manuscript, and update any in-text citations to match accordingly. Please see our Supporting Information guidelines for more information: http://journals.plos.org/plosone/s/supporting-information.

7 Please review your reference list to ensure that it is complete and correct. If you have cited papers that have been retracted, please include the rationale for doing so in the manuscript text, or remove these references and replace them with relevant current references. Any changes to the reference list should be mentioned in the rebuttal letter that accompanies your revised manuscript. If you need to cite a retracted article, indicate the article’s retracted status in the References list and also include a citation and full reference for the retraction notice.

[Note: HTML markup is below. Please do not edit.]

Reviewers' comments:

Reviewer's Responses to Questions

**Comments to the Author**

1. Is the manuscript technically sound, and do the data support the conclusions?

The manuscript must describe a technically sound piece of scientific research with data that supports the conclusions. Experiments must have been conducted rigorously, with appropriate controls, replication, and sample sizes. The conclusions must be drawn appropriately based on the data presented. 

Reviewer #1: Partly

Reviewer #2: Yes

**********

2. Has the statistical analysis been performed appropriately and rigorously? 

Reviewer #1: I Don't Know

Reviewer #2: Yes

**********

3. Have the authors made all data underlying the findings in their manuscript fully available?

The PLOS Data policy requires authors to make all data underlying the findings described in their manuscript fully available without restriction, with rare exception (please refer to the Data Availability Statement in the manuscript PDF file). The data should be provided as part of the manuscript or its supporting information, or deposited to a public repository. For example, in addition to summary statistics, the data points behind means, medians and variance measures should be available. If there are restrictions on publicly sharing data—e.g. participant privacy or use of data from a third party—those must be specified.

Reviewer #1: Yes

Reviewer #2: Yes

**********

4. Is the manuscript presented in an intelligible fashion and written in standard English?

PLOS ONE does not copyedit accepted manuscripts, so the language in submitted articles must be clear, correct, and unambiguous. Any typographical or grammatical errors should be corrected at revision, so please note any specific errors here.

Reviewer #1: Yes

Reviewer #2: Yes

**********

5. Review Comments to the Author

Please use the space provided to explain your answers to the questions above. You may also include additional comments for the author, including concerns about dual publication, research ethics, or publication ethics. (Please upload your review as an attachment if it exceeds 20,000 characters)

Reviewer #1: I’ve read with interest the draft “Association of ideal cardiovascular health metrics with incident chronic 2 kidney disease: more than a decade follow-up in the Tehran Lipid and 3 Glucose Study (TLGS)”, by Fatemeh Alizadeh, Maryam Tohidi, Mitra Hasheminia, Firoozeh Hosseini-Esfahani, Fereidoun Azizi, Farzad Hadaegh

However, some important issues have to be raised.

Introduction:

- Line 46-47. Authors should briefly introduce and define the main non-communicable diseases (NCDs).

- Line 65-66. A multifactorial treatment aimed at multiple risk factors reduces the risk of cardiovascular events in populations with type 2 diabetes mellitus and renal impairment. This issue is addressed in some recent literature which should be added to the references. Gaede P et al, N Engl J Med. 2003;348(5):383-393. doi:10.1056/NEJMoa021778; Sasso FC et al Cardiovasc Diabetol. 2021;20(1):145. Published 2021 Jul 16. doi:10.1186/s12933-021-01343-1

Methods and Materials:

- Line 128-129. Font typing error.

Discussion:

- Line 251. “china” is a typing error.

- Line 237-239. “Among middle-aged participants, the intermediate and ideal categories of BMI and BP, and the ideal category of FPG and in elderly individuals, only intermediate and ideal BP was associated with reduced risk of CKD”. The authors should better clarify the pathophysiological mechanisms/hypotheses supporting this sentence.

- Line 262. “Actually, no association was demonstrated between ICVHM and incident CKD among elder population”. It therefore seems that ICVHM does not affect the prognosis of elderly patients. However, some recent literature seems in contrast with this conclusion. The authors should better clarify the pathophysiological mechanisms/hypotheses supporting this sentence.

Reviewer #2: I read with great interest the paper “Referral pattern to nephrologist and prognosis in diabetic kidney disease patients: single center retrospective cohort study " by Iwata et al.

The article is well written. Paper design is fine. The article is logically divided into sections and subsections.

Comments:

1. Introduction: Diabetic nephropathy should be addressed to the right specialist as soon as possible to start the appropriate therapy and follow-up, in order to slow the disease progression. However, previous data have reported that despite the worst renal and cardiovascular prognosis, this subset of patients are at high risk of being under-treated independently of the type of clinical setting (nephrology, diabetology and primary care) (doi: 10.1097/01.hjh.0000239303.93872.31). This is also in contrast with the new view point of the multifactorial intervention in order to reduce the end stage kidney disease progression, but, even more importantly the cardiovascular risk factors. In fact, most of diabetic patients do not develop end stage kidney disease, as they die before (doi: 10.1186/s12933-021-01343-1).

2. Discussion, line 25-27: the authors reported that in their population the proteinuria levels are higher than in previous study, with a rapid decline in renal function. In these patients I would have also expected an increase in diabetic retinopathy onset with a consequent increase in cardiovascular events (doi: 10.1016/j.numecd.2019.05.065). Moreover, the authors reported after a line that renin angiotensin system inhibitors are used in this patients to reduce proteinuria, which is true, but with the use of this drugs we first assist to a reduction in glomerular filtration rate and after a stabilization in time, which may be compatible with a rapid decline in kidney function. Moreover, the role of SGLT2 inhibitors has been spread in this population, with an amelioration of both proteinuria and kidney function, together with a reduction of cardiovascular events, also representing the future, and not reported in the article (doi: 10.31083/j.rcm2303106).

**********

6. PLOS authors have the option to publish the peer review history of their article (what does this mean?). If published, this will include your full peer review and any attached files.

If you choose “no”, your identity will remain anonymous but your review may still be made public.

**Do you want your identity to be public for this peer review?** For information about this choice, including consent withdrawal, please see our Privacy Policy.

Reviewer #1: No

Reviewer #2: No

**********

[NOTE: If reviewer comments were submitted as an attachment file, they will be attached to this email and accessible via the submission site. Please log into your account, locate the manuscript record, and check for the action link "View Attachments". If this link does not appear, there are no attachment files.]

While revising your submission, please upload your figure files to the Preflight Analysis and Conversion Engine (PACE) digital diagnostic tool, https://pacev2.apexcovantage.com/. PACE helps ensure that figures meet PLOS requirements. To use PACE, you must first register as a user. Registration is free. Then, login and navigate to the UPLOAD tab, where you will find detailed instructions on how to use the tool. If you encounter any issues or have any questions when using PACE, please email PLOS at figures@plos.org. Please note that Supporting Information files do not need this step.

# Author comment: Author response to Decision Letter 0

**DOI:** 10.1371/journal.pone.0282163.r002

2 Dec 2022

Dr. Ferdinando Carlo Sasso

Academic Editor

Plos one

December 1, 2022

Dear Dr Sasso,

We wish to express our strong appreciation to the reviewers for their insightful comments on our paper entitled, “Referral pattern to nephrologist and prognosis in diabetic kidney disease patients: single center retrospective cohort study.”. We feel the comments have helped us significantly improve the manuscript.

We have incorporated changes that reflect the detailed suggestions you have graciously provided.

We have marked the changes highlighted in yellow in the text of the revised paper. 

We hope that the revisions in the manuscript and our accompanying responses will be sufficient to make our manuscript suitable for publication in Plos one.

Journal Requirements:

1. Please ensure that your manuscript meets PLOS ONE's style requirements, including those for file naming. The PLOS ONE style templates can be found at 

https://journals.plos.org/plosone/s/file?id=wjVg/PLOSOne_formatting_sample_main_body.pdf and 

https://journals.plos.org/plosone/s/file?id=ba62/PLOSOne_formatting_sample_title_authors_affiliations.pdf

Thank you for your comments. We modified our manuscript and supportive files. 

2. Please amend your current ethics statement to address the following concerns:

a) Did participants provide their written or verbal informed consent to participate in this study?

b) If consent was verbal, please explain i) why written consent was not obtained, ii) how you documented participant consent, and iii) whether the ethics committees/IRB approved this consent procedure.

c) If you are reporting a retrospective study of medical records or archived samples, please ensure that you have discussed whether all data were fully anonymized before you accessed them and/or whether the IRB or ethics committee waived the requirement for informed consent. If patients provided informed written consent to have data from their medical records used in research, please include this information.

We appreciate your comment on this point. Accordingly, we have added the following text to the Method section (page. 5, line 4): All data were fully anonymized before we accessed them and ethics committee waived the requirement for informed consent.

3. Thank you for stating the following in the Acknowledgments Section of your manuscript: 

"This research was supported in part by grant from the Osaka Kidney Foundation (OKF18-0001)."

We note that you have provided funding information that is not currently declared in your Funding Statement. However, funding information should not appear in the Acknowledgments section or other areas of your manuscript. We will only publish funding information present in the Funding Statement section of the online submission form. 

Please remove any funding-related text from the manuscript and let us know how you would like to update your Funding Statement. Currently, your Funding Statement reads as follows: 

"Y.I received grant from Osaka Kidney Foundation (http://www.okf.ecnet.jp/). Grant number is OKF18-0001.

The funders had no role in study design, data collection and analysis, decision to publish, or preparation of the manuscript."

Please include your amended statements within your cover letter; we will change the online submission form on your behalf. 

Thank you very much for your comments. We removed the Acknowledge section from manuscript. I would like to update my Finding statement as follows: 

"Y.I. received grant from Osaka Kidney Foundation (http://www.okf.ecnet.jp/). Grant number is OKF18-0001.

The funders had no role in study design, data collection and analysis, decision to publish, or preparation of the manuscript."

4. PLOS requires an ORCID iD for the corresponding author in Editorial Manager on papers submitted after December 6th, 2016. Please ensure that you have an ORCID iD and that it is validated in Editorial Manager. To do this, go to ‘Update my Information’ (in the upper left-hand corner of the main menu), and click on the Fetch/Validate link next to the ORCID field. This will take you to the ORCID site and allow you to create a new iD or authenticate a pre-existing iD in Editorial Manager. Please see the following video for instructions on linking an ORCID iD to your Editorial Manager account: https://www.youtube.com/watch?v=_xcclfuvtxQ

Thank you for your comment. In accordance with your comment, we have ensured ORCID ID.

5. Please amend either the title on the online submission form (via Edit Submission) or the title in the manuscript so that they are identical.

We appreciate your suggestion. We have changed title as follows: Referral pattern to nephrologist and prognosis in diabetic kidney disease patients: single center retrospective cohort study 

6. Please include captions for your Supporting Information files at the end of your manuscript, and update any in-text citations to match accordingly. Please see our Supporting Information guidelines for more information: http://journals.plos.org/plosone/s/supporting-information.

Thank you for your comments. Accordingly, we added Supporting information files at the end of our manuscript including captions. 

7 Please review your reference list to ensure that it is complete and correct. If you have cited papers that have been retracted, please include the rationale for doing so in the manuscript text, or remove these references and replace them with relevant current references. Any changes to the reference list should be mentioned in the rebuttal letter that accompanies your revised manuscript. If you need to cite a retracted article, indicate the article’s retracted status in the References list and also include a citation and full reference for the retraction notice.

Thank you for your suggestion. We modified our reference list. We removed one reference paper: Imai E, et al. CKD clinical practice guideline 2012. Nihon Jinzo Gakkai shi. 2012; 54: 1031-191.

In addition, we added seven papers to reference section: Sasso FC, Pafundi PC, Simeon V, Nicola LD, Chiodini P, Galiero R, et al. Efficacy and durability of multifactorial intervention on mortality and MACEs: a randomized clinical trial in type-2 diabetic kidney disease. Cardiovasc Diabetol. 2021; 20: 145, Ueki K, Sasako T, Okazaki Y, Miyake K, Nangaku M, Ohashi Y, et al. Multifactorial intervention has a significant effect on diabetic kidney disease in patients with type 2 diabetes. Kidney Int. 2021; 99; 256-266, Minutolo R, Sasso FC, Chiodini P, Cianciaruso B, Carbonara O, Zamboli P, et al. Management of cardiovascular risk factors in advanced type 2 diabetic nephropathy: a comparative analysis in nephrology, diabetology and primary care settings. J Hypertens. 2006; 24; 1655-61, Sasso FC, Pafundi PC, Gelso A, Bono V, Costagliola C, Marfella R, et al. Relationship between albuminuric CKD and diabetic retinopathy in a real-world setting of type 2 diabetes: Findings from No blind study. Nutr Metab Cardiovasc Dis. 2019; 29: 923-930, Naresh CN, Hayen A, Weening A, Craig JC, Chadban SJ. Day-to-day variability in spot urine albumin-creatinine ratio. Am J Kidney Dis. 2013; 62: 1095-101, Tanaka K, Watanabe T, Takeuchi A, Ohashi Y, Nitta K, Akizawa T, et al. Cardiovascular events and death in Japanese patients with chronic kidney disease. Kidney Int. 2017; 91: 227-234 and Nevola R, Alfano M, Pafundi PC, Brin C, Gragnano F, Calabrò P, et al. Cardiorenal Impact of SGLT-2 Inhibitors: A Conceptual Revolution in The Management of Type 2 Diabetes, Heart Failure and Chronic Kidney Disease. Rev Cardiovasc Med. 2022; 23; 106.

Therefore, we correct the number of reference list. 

Review Comments to the Author

Reviewer #2: I read with great interest the paper “Referral pattern to nephrologist and prognosis in diabetic kidney disease patients: single center retrospective cohort study " by Iwata et al.

The article is well written. Paper design is fine. The article is logically divided into sections and subsections.

Comments:

1. Introduction: Diabetic nephropathy should be addressed to the right specialist as soon as possible to start the appropriate therapy and follow-up, in order to slow the disease progression. However, previous data have reported that despite the worst renal and cardiovascular prognosis, this subset of patients are at high risk of being under-treated independently of the type of clinical setting (nephrology, diabetology and primary care) (doi: 10.1097/01.hjh.0000239303.93872.31). This is also in contrast with the new view point of the multifactorial intervention in order to reduce the end stage kidney disease progression, but, even more importantly the cardiovascular risk factors. In fact, most of diabetic patients do not develop end stage kidney disease, as they die before (doi: 10.1186/s12933-021-01343-1).

We appreciate your comments on this point. We have added and modified the following text to the introduction section (page. 3, line 14): On the other hand, most of diabetic patients do not develop ESKD, as they die before [2]. Therefore, management of DKD to prevent ESKD and improve overall survival has become a major challenge in public health and clinical medicine. 

In addition, we have added the sentences in the introduction section (page. 4, line 6): Although multifactorial intervention significantly reduced the onset or progression of DKD in patients with type 2 diabetes mellitus [19], patients with advanced DKD, despite the worst renal and cardiovascular prognosis, are at high risk of being under-treated independently of the type of clinical setting (nephrology, diabetology and primary care) [20].

2. Discussion, line 25-27: the authors reported that in their population the proteinuria levels are higher than in previous study, with a rapid decline in renal function. In these patients I would have also expected an increase in diabetic retinopathy onset with a consequent increase in cardiovascular events (doi: 10.1016/j.numecd.2019.05.065). Moreover, the authors reported after a line that renin angiotensin system inhibitors are used in this patients to reduce proteinuria, which is true, but with the use of this drugs we first assist to a reduction in glomerular filtration rate and after a stabilization in time, which may be compatible with a rapid decline in kidney function. Moreover, the role of SGLT2 inhibitors has been spread in this population, with an amelioration of both proteinuria and kidney function, together with a reduction of cardiovascular events, also representing the future, and not reported in the article (doi: 10.31083/j.rcm2303106).

We appreciate your suggestion on this point. Regrettably, we did not retrieve the information from all patient’s records whether patients had the diabetic retinopathy or not because this is retrospective study. We added the sentences to the discussion section (page. 18, line 6); It was also reported that diabetic retinopathy could predict subsequent cardiovascular events in patients with diabetes mellitus [30]; however, due to the retrospective nature of this study, we didn't have enough data on diabetic retinopathy, so we could not investigate the association between diabetic retinopathy and CVE onset.

Although about 70% of our patients already used renin angiotensin system inhibitors at nephrology referral, we did not investigate the treatment details including medications after nephrology referral. So we modified the sentences in discussion section (page. 21, line 6): Second, we did not investigate the treatment details after the nephrology referral, such as blood pressure, fluid and mineral levels, bone disorders, anemia, metabolic acidosis, glycemic control and medications.

In response to reviewer’s comment, we modified the sentence in the discussion section (page. 20, lines 11); However, because these studies excluded patients with eGFR of 25–30 mL/min/1.73 m2 or less and furthemore, in the present study, SGLT2 inhibitors were not widely used, so it is not clear whether these agents will improve the cardiovascular outcomes in patients with advanced stages of DKD. Early nephrology referral may improve both renal and cardiovascular outcomes.

Yours sincerely,

Yukimasa Iwata

Department of Kidney Disease and Hypertension, Osaka General Medical Center

3-1-56 Bandaihigashi, Sumiyoshi-ku, Osaka 558-8558, Japan

Tel: +81-6-6692-1201

Fax: +81-6-6606-7000

E-mail: yuki_iwata0718@yahoo.co.jp

Attachment Submitted filename: Response to Reviewers.docx Click here for additional data file.

# Peer review: Decision Letter 1

**DOI:** 10.1371/journal.pone.0282163.r003

**Authors:** Ferdinando Carlo Sasso

9 Feb 2023

Referral pattern to nephrologist and prognosis in diabetic kidney disease patients: single center retrospective cohort study

PONE-D-22-18940R1

Dear Dr. Hayashi,

We’re pleased to inform you that your manuscript has been judged scientifically suitable for publication and will be formally accepted for publication once it meets all outstanding technical requirements.

Within one week, you’ll receive an e-mail detailing the required amendments. When these have been addressed, you’ll receive a formal acceptance letter and your manuscript will be scheduled for publication.

An invoice for payment will follow shortly after the formal acceptance. To ensure an efficient process, please log into Editorial Manager at http://www.editorialmanager.com/pone/, click the 'Update My Information' link at the top of the page, and double check that your user information is up-to-date. If you have any billing related questions, please contact our Author Billing department directly at authorbilling@plos.org.

If your institution or institutions have a press office, please notify them about your upcoming paper to help maximize its impact. If they’ll be preparing press materials, please inform our press team as soon as possible -- no later than 48 hours after receiving the formal acceptance. Your manuscript will remain under strict press embargo until 2 pm Eastern Time on the date of publication. For more information, please contact onepress@plos.org.

Kind regards,

Ferdinando Carlo Sasso, PhD, MD

Academic Editor

PLOS ONE

Additional Editor Comments (optional):

No further comments

Reviewers' comments:

Reviewer's Responses to Questions

**Comments to the Author**

1. If the authors have adequately addressed your comments raised in a previous round of review and you feel that this manuscript is now acceptable for publication, you may indicate that here to bypass the “Comments to the Author” section, enter your conflict of interest statement in the “Confidential to Editor” section, and submit your "Accept" recommendation.

Reviewer #1: All comments have been addressed

Reviewer #2: All comments have been addressed

**********

2. Is the manuscript technically sound, and do the data support the conclusions?

The manuscript must describe a technically sound piece of scientific research with data that supports the conclusions. Experiments must have been conducted rigorously, with appropriate controls, replication, and sample sizes. The conclusions must be drawn appropriately based on the data presented. 

Reviewer #1: Yes

Reviewer #2: Yes

**********

3. Has the statistical analysis been performed appropriately and rigorously? 

Reviewer #1: Yes

Reviewer #2: Yes

**********

4. Have the authors made all data underlying the findings in their manuscript fully available?

The PLOS Data policy requires authors to make all data underlying the findings described in their manuscript fully available without restriction, with rare exception (please refer to the Data Availability Statement in the manuscript PDF file). The data should be provided as part of the manuscript or its supporting information, or deposited to a public repository. For example, in addition to summary statistics, the data points behind means, medians and variance measures should be available. If there are restrictions on publicly sharing data—e.g. participant privacy or use of data from a third party—those must be specified.

Reviewer #1: Yes

Reviewer #2: Yes

**********

5. Is the manuscript presented in an intelligible fashion and written in standard English?

PLOS ONE does not copyedit accepted manuscripts, so the language in submitted articles must be clear, correct, and unambiguous. Any typographical or grammatical errors should be corrected at revision, so please note any specific errors here.

Reviewer #1: Yes

Reviewer #2: Yes

**********

6. Review Comments to the Author

Please use the space provided to explain your answers to the questions above. You may also include additional comments for the author, including concerns about dual publication, research ethics, or publication ethics. (Please upload your review as an attachment if it exceeds 20,000 characters)

Reviewer #1: (No Response)

Reviewer #2: The author managed to answer to all my queries. The paper has much improved and can be further processed for publication.

**********

7. PLOS authors have the option to publish the peer review history of their article (what does this mean?). If published, this will include your full peer review and any attached files.

If you choose “no”, your identity will remain anonymous but your review may still be made public.

**Do you want your identity to be public for this peer review?** For information about this choice, including consent withdrawal, please see our Privacy Policy.

Reviewer #1: No

Reviewer #2: No

**********

# Editor report: Acceptance letter

**DOI:** 10.1371/journal.pone.0282163.r004

**Authors:** Ferdinando Carlo Sasso

14 Feb 2023

PONE-D-22-18940R1 

Referral pattern to nephrologist and prognosis in diabetic kidney disease patients: single center retrospective cohort study 

Dear Dr. Hayashi:

I'm pleased to inform you that your manuscript has been deemed suitable for publication in PLOS ONE. Congratulations! Your manuscript is now with our production department. 

If your institution or institutions have a press office, please let them know about your upcoming paper now to help maximize its impact. If they'll be preparing press materials, please inform our press team within the next 48 hours. Your manuscript will remain under strict press embargo until 2 pm Eastern Time on the date of publication. For more information please contact onepress@plos.org.

If we can help with anything else, please email us at plosone@plos.org. 

Thank you for submitting your work to PLOS ONE and supporting open access. 

Kind regards, 

PLOS ONE Editorial Office Staff

on behalf of

Professor Ferdinando Carlo Sasso 

Academic Editor

PLOS ONE
